# Hypoxic conditioned medium from mesenchymal stem cells promotes lymphangiogenesis by regulation of mitochondrial-related proteins

**DOI:** 10.1186/s13287-016-0296-1

**Published:** 2016-03-11

**Authors:** Chang Youn Lee, Jin Young Kang, Soyeon Lim, Onju Ham, Woochul Chang, Dae-Hyun Jang

**Affiliations:** Department of Integrated Omics for Biomedical Sciences, Graduate School, Yonsei University, Seoul, 03722 Republic of Korea; Department of Rehabilitation Medicine, National Traffic Injury Rehabilitation Hospital, College of Medicine, The Catholic University of Korea, Yangpyeong-gun, 12564 Republic of Korea; Institute for Bio-Medical Convergence, College of Medicine, Catholic Kwandong University, Gangneung-si, 25601 Gangwon-do Republic of Korea; Catholic Kwandong University International St. Mary’s Hospital, Incheon Metropolitan City, 22711 Republic of Korea; Department of Biology Education, College of Education, Pusan National University, Busan, 46241 Republic of Korea; Department of Rehabilitation Medicine, Incheon St. Mary’s Hospital, College of Medicine, The Catholic University of Korea, Dongsu-ro 56, Bupyeong-gu, Incheon 21431 Republic of Korea

**Keywords:** Hypoxic conditioned media, Mesenchymal stem cells, Mitochondrial-related protein, Lymphangiogenesis, Lymphatic endothelial cells

## Abstract

**Background:**

Recently, cell-based therapeutic lymphangiogenesis has emerged and provided hope for lymphatic regeneration. Previous studies have demonstrated that secretomes of mesenchymal stem cells (MSCs) facilitate the regeneration of various damaged tissues. This study was conducted to evaluate the lymphangiogenic potential of hypoxic conditioned media (HCM) from MSCs.

**Methods:**

To investigate the effects of MSC-secreted factors in starved human lymphatic endothelial cells (hLEC), hLECs were treated with endothelial basal medium (EBM)-2 (control), normoxic conditioned media (NCM), or HCM *in vitro* and *in vivo.*

**Results:**

MSCs expressed lymphangiogenic factors including EGF, FGF2, HGF, IGF-1, and VEGF-A and -C. hLECs were treated with each medium. hLEC proliferation, migration, and tube formation were improved under HCM compared with NCM. Moreover, expression of mitochondrial-related factors, MFN1and 2, were improved in HCM-treated hLECs. Lymphedema mice injected with HCM showed markedly decreased lymphedema via increased lymphatic vessel formation when compared with EBM-2- or NCM-treated mice.

**Conclusions:**

This study suggested that HCM from MSCs contain high levels of secreted lymphangiogenic factors and promote lymphangiogenesis by regulating mitochondrial-related factors. Thus, treatment with HCM may be a therapeutic strategy for lymphedema.

## Background

Lymphedema is a pathologic swelling (edema) that results from the accumulation of protein-rich fluid in the interstitial space because of congenital or acquired lymphatic system damage [1]. Secondary lymphedema is caused by disruption or obstruction of the normal lymphatic system in response to infection, trauma, or iatrogenic processes such as surgery (radical lymph node dissection) or radiation-related cancer therapy [2]. Secondary lymphedema is a lifelong condition that disturbs quality of life with signs and symptoms such as heaviness, discomfort, and impaired mobility of the limbs as well as a weakened immunity. To date, the only proposed treatment options have been pharmacotherapy, physiotherapy, and surgical treatments, such as lymph node transfer and lymphatic bypass. However, these methods require good compliance and lifelong care [3]. Recently, cell-based therapy for therapeutic lymphangiogenesis has emerged and made lymphatic regeneration possible [4]. Essential lymphatic growth factors, including epidermal growth factor (EGF), fibroblast growth factor (FGF), hepatocyte growth factor (HGF), insulin-like growth factor (IGF)-1, vascular endothelial growth factor (VEGF)-A, and VEGF-C, have been identified in several studies [5–10]. In addition, these growth factors have been shown to contribute to lymphangiogenesis in the damaged lymphatic areas [11].

Mesenchymal stem cells (MSCs) are multipotent cells that can be obtained from adult donors and are known to have a low risk of immune rejection [12]. Therefore, therapeutic applications using MSCs have been extensively studied and applied to various tissues in regenerative medicine owing to these beneficial effects. Moreover, MSC therapy has recently been introduced to the market. However, limitations such as poor survival, limited differentiation, and dedifferentiation of cells with passaging and donor site morbidity exist [13–16]. To avoid these limitations, researchers have turned their attention to new therapeutic approaches. MSCs are known to secrete various cytokines and growth factors, which show paracrine and autocrine activities for injured cells, particularly for those in hypoxic, apoptotic, or inflamed areas [17, 18]. The secreted factors have been demonstrated to have many beneficial therapeutic effects in various diseases, such as neurodegenerative diseases, cancers, and heart failure [19]. Several reports have shown that cytokines secreted from MSCs under normal growth conditions can promote lymphangiogenesis via VEGF-A and VEGF-C [20–22]. MSC-based therapy has been suggested to be the most promising stem cell therapy for lymphangiogenesis [4].

Some researchers showed the possibility of regulating stem cell paracrine actions via different culture methods [18]. As one of the modifications, MSCs exposed to hypoxia showed more protein secretion and greater paracrine effects. MSCs under hypoxia also showed increased proliferation and migration compared with those under normal growth conditions, and treatment with conditioned media from hypoxic MSCs exerted therapeutic effects on wound healing by enhancing the production of angiogenic paracrine factors including basic FGF, IGF, and VEGF [23]. Another study reported that conditioned media obtained from MSCs under hypoxia showed protective effects against cardiomyoblasts in hypoxia and angiogenic effects on endothelial cells [24].

Mitochondria are important organelles that maintain cellular homeostasis under stressful conditions, such as apoptotic stimuli, an increase in reactive oxygen species (ROS), and changes in intracellular calcium concentration [25–27]. To maintain their function, mitochondria continuously undergo fission and fusion. Mitofusins MFN1 and MFN2 in the mitochondrial outer membrane have been shown to cause mitochondrial membrane fusion by binding with OPA1 in the inner membrane, whereas dynamin-related protein 1 is mainly involved with mitochondrial fission based on the phosphorylation status [28, 29]. Recent studies demonstrated that MFNs are necessary for angiogenic function in endothelial cells [30, 31]. In addition, VEGF-A is an important factor for angiogenesis, which can stimulate the activation of MFN-mediated signaling pathways [31]. We hypothesized that MFNs also play important roles in lymphangiogenesis.

We investigated the therapeutic ability of hypoxic conditioned media (HCM) from MSCs for lymphatic edema using in vitro and in vivo experimental systems. Higher expression of VEGF, a lymphangiogenic factor, was observed in hypoxic MSCs, and tube formation increased in human lymphatic endothelial cells (hLECs) treated with HCM. Overall, we confirmed that HCM have the ability to induce lymphangiogenesis in hLECs and electrocauterized mice.

## Methods

### Cell culture

Human bone marrow MSCs (hMSCs; catalogue number PT-2501) and adult human dermal lymphatic microvascular endothelial cells (hLECs) (HMVECs-dLyAd; catalogue number CC-2810) were purchased from Lonza (Basel, Switzerland). hMSCs were maintained at 37 °C in a humidified atmosphere containing 5 % CO_2_. Culture media was composed of 10 % fetal bovine serum (Invitrogen, Waltham, MA, USA), Dulbecco’s modified Eagle’s medium—low glucose, 100 U/ml penicillin (Invitrogen), and 100 μg/ml streptomycin (Invitrogen). Media were replaced every 3 days. We used 7–10 passages of hMSCs for experiments. hLECs were cultured in Lonza EGM-2MV medium and replaced with fresh media every 2 days.

### HCM from MSCs

hMSCs were incubated for 1 day after they were seeded in a 100 mm dish (1 × 10^6^ cells/dish), washed twice with endothelial basal medium (EBM-2; Lonza), and then placed into a hypoxic chamber (Anaerobic Environment; ThermoForma, Waltham, MA, USA) containing 5 ml EBM-2 for 12 hours. The airtight humidified hypoxic chamber was maintained at 37 °C and continuously supplied with mixed gas (5 % CO_2_, 10 % H_2_, and 85 % N_2_). The oxygen level in the chamber was ~0.5 %. Following incubation, the medium was collected and centrifuged at 1000 × *g* for 10 minutes at 4 °C, after which the supernatant was transferred to a new tube. Similarly, normoxic conditioned medium (NCM) was derived from hMSC-cultured media under normoxic conditions for 12 hours with 5 ml EBM-2. Each medium was stored at −80 °C until used.

### Lymphatic endothelial cells

#### Proliferation assay

Cell proliferation was measured using a Cell Counting Kit-8 (Dojindo, Kumamoto, Japan). hLECs were seeded at 5 × 10^3^ cells per well in 96-well culture plates and then cultured for 1 day. hLECs were then incubated with fresh EBM-2 for 12 hours. After the medium was removed, cells were washed twice with phosphate-buffered saline (PBS). Each group was subsequently treated with EBM-2, NCM, or HCM, respectively, after which the cells were incubated for 24 hours in a 37 °C humidified atmosphere incubator containing 5 % CO_2_. Following incubation, Cell Counting Kit-8 was added to each well and samples were then incubated for 2 hours. Finally, the absorbance of water soluble formazan dye was measured at 450 nm using a microplate reader (Molecular Devices, Sunnyvale, CA, USA). All experiments were performed in triplicate.

#### Migration assay

hLECs (2 × 10^4^ cells) were seeded into the upper chamber of a Transwell filter with 8 μm pores (Costar Corning, New York, NY, USA) coated with 10 μg/ml fibronectin. They were deprived of serum for 12 hours with EBM-2, after which EBM-2, NCM, or HCM were added to the lower chamber. Cells on the upper chambers were incubated at 37 °C for 9 hours under different stimuli. Following incubation, cells on the underside of the filter were stained with 0.25 % crystal violet. Nonmigrating cells on the upper side of the filter were removed with cotton swabs. The filter was then photographed using a digital microscope camera system (Olympus, Shinjuku, Japan), or stained cells were dissolved in 10 % acetic acid and transferred to a 96-well plate for colorimetric reading at 560 nm using a microplate reader (Molecular Devices).

#### Immunoblot analysis

hLECs were washed twice in ice-cold PBS, then lysed with lysis buffer (Cell Signaling, Danvers, MA, USA) containing the protease inhibitor cocktail PhosSTOP (Roche, Basel, Switzerland), and incubated at 4 °C for 30 minutes. Protein concentrations were determined using a BCA protein assay kit (Thermo Fisher Scientific, Inc., Waltham, MA, USA), after which they were separated in 10 % SDS–polyacrylamide gel and transferred to PVDF membrane (Millipore, Billerica, MA, USA). The membrane was then blocked using TBS-T (0.1 % Tween 20) containing 5 % (w/v) bovine serum albumin (BSA) for 1 hour at room temperature. The membranes were then washed with TBS-T and incubated with primary antibody overnight at 4 °C. The next morning, the membrane was washed three times with TBS-T for 5 minutes each and incubated with horseradish peroxidase-conjugated secondary antibody for 1 hour at room temperature. After extensive washing, bands were detected using enhanced chemiluminescence reagent (AbClon, Seoul, Republic of Korea) and band intensities were measured using a Photo-Image System (Molecular Dynamics, Sunnyvale, CA, USA). MFN1 (Novus, Littleton, CO, USA), MFN2 (Sigma, St. Louis, MO, USA), extracellular signal regulated kinase (ERK; Santa Cruz, CA, USA), p-ERK (Santa Cruz), lymphatic vessel endothelial hyaluronan receptor 1(LYVE-1; Novus), and beta-actin (Sigma) antibodies were used in the experiment.

#### Real-time PCR

Total RNA was isolated using TRIzol® Reagent (Life Technologies, Waltham, MA, USA). The total RNA quality and concentration were measured using NanoDrop Lite (Thermo Fisher Scientific, Inc.). Single-stranded cDNA was synthesized from total RNA using a reverse transcription system (Promega, Fitchburg, WI, USA) according to the product guidelines. Amplification and detection of specific products were performed in a StepOnePlus Real-time PCR System (Life Technologies) using a FastStart Essential DNA Green Master (Roche). PCR conditions consisted of 95 °C for 10 minutes followed by 40 cycles of 95 °C for 10 seconds and 60 °C for 10 seconds. The threshold cycle (Ct) of each target gene was automatically defined and normalized to the control glyceraldehyde 3-phosphate dehydrogenase (GAPDH) (ΔCt value). The relative differences in the expression levels of each mRNA in VSMCs (^ΔΔ^Ct) were calculated and presented as fold induction (2^−ΔΔ^Ct). Real-time PCR primers consisted of the following groups: EGF forward primer, 5′-GGT CTT GCT GTG GAC TGG AT-3′; EGF reverse primer, 5′-CTG CTA CAG CAA ATG GGT GA-3′; IGF-1 forward primer, 5′-TCA CCT TCA CCA GCT CTG C-3′; IGF-1 reverse primer, 5′-TGG TAG ATG GGG GCT GAT AC-3′; HGF forward primer, 5′-GCC TGA AAG ATA TCC CGA CA-3′; HGF reverse primer, 5′-GCC ATT CCC ACG ATA ACA AT-3′; FGF-2 forward primer, 5′-AGC GGC TGT ACT GCA AAA AC-3′; FGF reverse primer, 5′-CTT TCT GCC CAG GTC CTG TT-3′; VEGF-A forward primer, 5′-AGT CCA ACA TCA CCA TGC AG-3′; VEGF-A reverse primer, 5′-TTC CCT TTC CTC GAA CTG ATT T-3′; VEGF-C forward primer, 5′-CCT CAA CTC AAG GAC AGA AGA G-3′; VEGF-C reverse primer, 5′-CTG GCA GGG AAC GTC TAA TAA T-3′; GAPDH forward primer, 5′-ACA TCG CTC AGA CAC CAT G-3′; and GAPDH reverse primer, 5′-TGT AGT TGA GGT CAA TGA AGG G-3′.

#### Tube formation assay

A Matrigel-based tube formation assay was performed. Each well of a 96-well culture plate was coated with 50 μl of ECMatrix™ (Millipore) and then allowed to incubate for 1 hour at 37 °C. Next, hLECs were seeded onto the coated wells at a density of 1 × 10^4^ cells/well, cultured with 500 μl of EBM-2, NCM, or HCM, and incubated at 37 °C under 5 % CO_2_ for 12 hours. Following incubation, tube formation images were captured using a digital microscope camera system (Olympus).

### Hind limb mouse model of lymphedema

The hind limb mouse model of lymphedema was obtained as described previously [11]. Eight-week-old BALB/c mice (Orient Bio Co., Seongnam, Korea) were assigned into groups of three. Normal mice were then anesthetized via subcutaneous injection of zoletil (4 mg/kg) with rompun (20 mg/kg). After a mouse was fully anesthetized, 0.5 % Evans blue solution was intradermally injected into the footpad of the hind limb to visualize the lymphatic vessels. A circumferential incision of the limb (thigh) was made to access lymph vessels that were subsequently electrocauterized (Bovie Medical Corporation, Clearwater, FL, USA). EBM-2, NCM, and HCM were then subcutaneously injected at the site of the damaged area on the second day after preparing the edema models and then every 3 days after that for a period of 24 days. The injection volume was 100 μl of 1-in-50 concentrated media. All animals were maintained under a 12-hour light–dark cycle condition and had free access to food and water. Experimental procedures were approved by the Committee for Care and Use of Laboratory Animals, Yonsei University College of Medicine, and performed in accordance with the Guidelines and Regulations for Animal Care.

### Histological analysis

To measure lymphangiogenesis, mice were sacrificed 4 weeks after the surgery, after which a mouse’s entire thigh region, including the site at which hMSC-conditioned medium was injected, was dissected. The hind limbs were excised from the sacrificed mouse, washed with PBS to remove the blood, and then fixed in 10 % formalin solution for 1 day at 4 °C. Samples were subsequently treated with decalcification solution (Calci-Clear Rapid; National Diagnostics, Atlanta, GA, USA) for 1 week. Tissue sections were sequentially mounted onto gelatin-coated glass slides to ensure that different stains could be used on successive tissue sections sliced through the injured area. After the sections were deparaffinized and rehydrated, samples were blocked using 2.5 % normal horse serum and incubated with an anti-LYVE-1 antibody. A biotinylated pan-specific universal secondary antibody and streptavidin/peroxidase complex reagent were used to treat the sections. Sections were subsequently stained with an antibody using a DAB substrate kit (Vector, Burlingame, CA, USA). Counterstaining was performed using 1 % methyl green, and dehydration was performed using 100 % n-butanol, ethanol, and xylene before mounting in VectaMount Mounting Medium (Vector, Burlingame, CA, USA). A coverslip was placed on top of each section, and the sections were then observed using light microscopy. The images for hematoxylin and eosin (H & E) and LYVE-1 were obtained using virtual microscopy (BX51/dot Slide; Olympus). Images for LYVE-1 were detected using microscopy and transferred to a computer equipped with the MetaMorph software (version 4.6; Molecular Devices, Sunnyvale, CA, USA).

### In vivo visualized lymphatic vessels

We observed the lymphatic vessels with fluorescence 3 weeks after surgery. The hind limb mouse model was anesthetized via a subcutaneous injection of zoletil (4 mg/kg) with rompun (20 mg/kg). After the mouse was fully anesthetized, 30 μl of fluorescein isothiocyanate (FITC)-dextran solution (1 mg/ml) was intradermally injected into the footpad of the hind limb to visualize the lymphatic vessels, after which the lymphatic vessels were observed under a fluorescence microscope (SteREO Discovery V12; Zeiss, Oberkochen, Germany).

### Statistical analysis

Data are expressed as mean ± standard deviation (SD). Significance differences between groups were identified using Student’s *t* test. Comparisons between more than two groups were made with one-way analysis of variance using Bonferroni’s correction. *p* <0.05 was considered statistically significant.

## Results

### HCM enhance lymphangiogenesis in hLECs

**Fig. 1:**
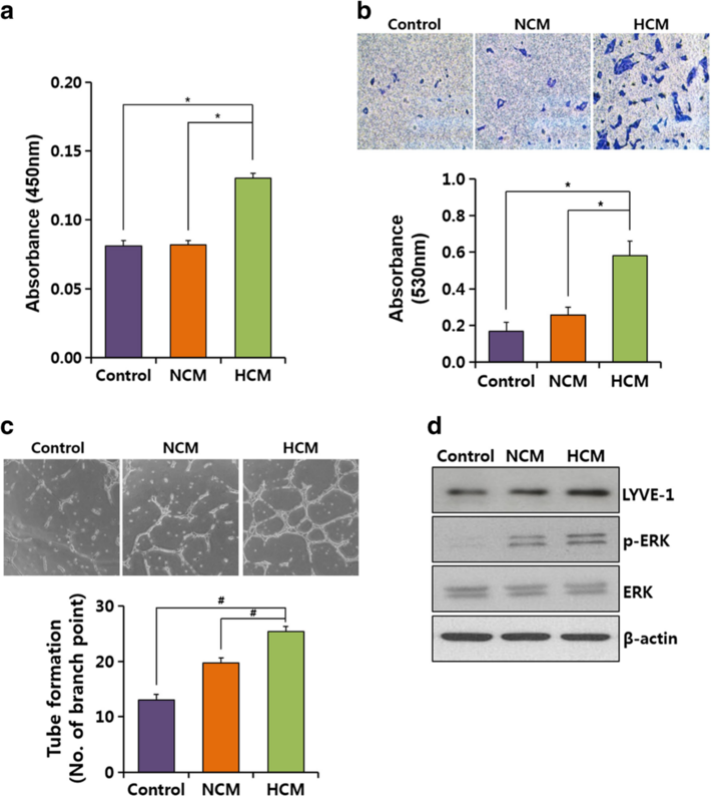
hMSC-derived HCM stimulate hLECs in vitro. **a** hLEC proliferation was measured using the Cell Counting Kit-8 assay. **b** Representative images showed the results of migration assays of hLECs treated with NCM and HCM. **c** Representative photographs of tube formation in hLECs after treatment with NCM and HCM. **d** hLEC specific marker, LYVE-1, and phosphorylation of ERK were detected using western blot analysis. Values are represented as the mean of three measurements with the SD indicated by error bars. Control: EBM. *p <0.001, ^#^ *p* <0.05. *ERK* extracellular signal regulated kinase, *GAPDH* glyceraldehyde 3-phosphate dehydrogenase, *HCM* hypoxic conditioned media, *LYVE-1* lymphatic vessel endothelial hyaluronan receptor 1, *NCM* normoxic conditioned media

To investigate the ability of HCM to undergo lymphangiogenesis in vitro, we tested the proliferation, migration, and tube formation in hLECs. Under normal growth conditions, HCM did not cause any positive effects and was similar to NCM (data not shown). Therefore, hLECs were starved in serum-free EBM-2 culture for 12 hours to simulate in vivo conditions prior to NCM or HCM treatment, after which the effects of HCM on lymphangiogenesis were investigated. HCM markedly increased proliferation by up to twofold (Fig. 1a), as well as migration (Fig. 1b), compared with the control media and NCM. In addition, tube formation was promoted with HCM (Fig. 1c). We also examined LYVE-1 and ERK phosphorylation as proliferation markers, resulting in increased expression and phosphorylation under HCM treatment (Fig. 1d). The results showed that HCM have the ability to induce lymphangiogenesis in hLECs.

### hMSCs induce the expression of pro-lymphangiogenesis factors under hypoxia

**Fig. 2:**
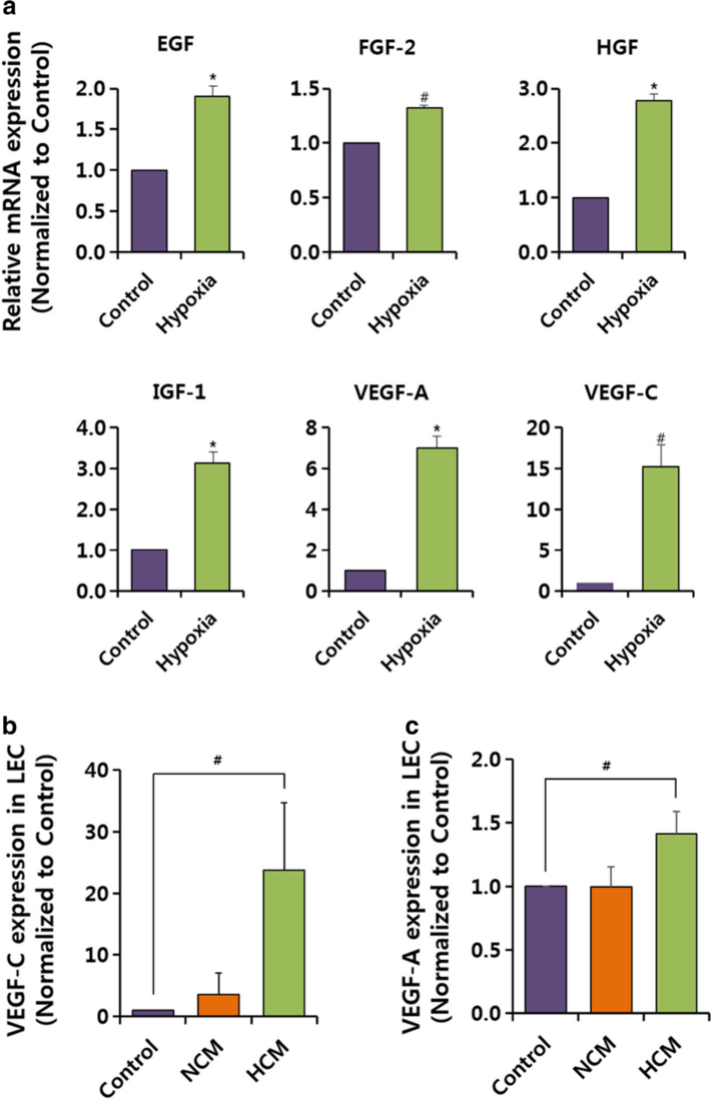
Increased expression of lymphangiogenic factors of hMSCs in hypoxia. **a** Expression of lymphangiogenic factors (EGF, FGF, HGF, IGF-1, VEGF-A, and VEGF-C) of hMSCs in hypoxia. Expression of mRNAs was measured using quantitative RT-PCR. Gene expressions were normalized using GAPDH expression, respectively. **b**, **c** Expression of VEGF-C and VEGF-A was analyzed using quantitative RT-PCR in EBM, NCM, or HCM-treated hLECs. Control: EBM. Values are represented as the mean of three measurements with the SD indicated by error bars. *p <0.001, ^#^ *p* <0.05. *EGF* epidermal growth factor, *FGF* fibroblast growth factor, *HCM* hypoxic conditioned media, *HGF* hepatocyte growth factor, *IGF* insulin-like growth factor, *LEC* lymphatic endothelial cells, *NCM* normoxic conditioned media, *VEGF* vascular endothelial growth factor

We next investigated several growth factors from hMSCs, which are known to induce lymphangiogenesis in hLECs. hMSCs exposed to 12 hours of hypoxia were lysed and used to estimate mRNA expression levels. The mRNA expression of six different pro-lymphangiogenesis factors increased, with VEGF-A and VEGF-C showing significant increases in gene expression (Fig. 2a). Therefore, we further examined whether the expression of VEGF-A and VEGF-C (important lymphangiogenic-stimulating factors) increased in response to HCM in hLECs. Both factors markedly increased under HCM compared with NCM, which is the medium normally used for hMSC cultivation (Fig. 2b, c).

### HCM affect the expression of MFN1/2 in hLECs

**Fig. 3:**
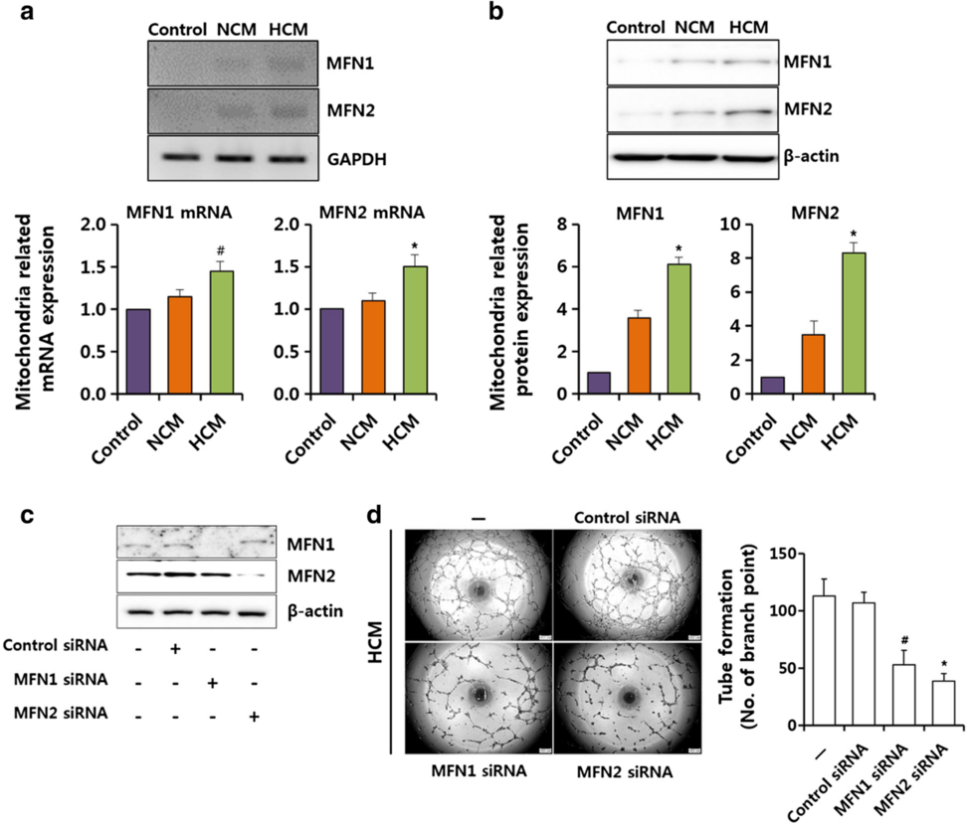
Effects of HCM on expression of mitochondria-related genes in hLECs. **a** hLECs were starved in EBM for 12 hours and then subjected to hMSC-conditioned medium treatment for 12 hours. MFN1 and MFN2 mRNA expression were analyzed using RT-PCR. mRNA expression was normalized using GAPDH. **b** hLECs were starved in EBM for 12 hours and then subjected to hMSC-conditioned medium treatment for 12 hours. MFN1 and MFN2 expression was measured using western blot analysis. **c** Expression of MFN1/2 was detected in MFN1/2 siRNA-transfected hLECs and the expression of MFN1/2 by immunoblotting. **d** Representative photographs were tube formation of MFN1/2 siRNA-transfected hLECs after treatment of each media. Control: EBM. Values are represented as the mean of three measurements with the SD indicated by error bars. *p <0.001, ^#^ *p* <0.05. *GAPDH* glyceraldehyde 3-phosphate dehydrogenase, *HCM* hypoxic conditioned media, *MFN* mitofusin, *NCM* normoxic conditioned media, *siRNA* small interfering RNA

VEGF is known to induce MFNs in vascular endothelial cells but not in hLECs. Therefore, we examined expression levels of mRNA and protein of MFNs in hLECs under different conditions. HCM-treated cells showed the ability to induce both MFN1 and MFN2 expression compared with NCM-treated cells. NCM also increased MFN1 and MFN2 expression relative to the basal levels of these genes and proteins (Fig. 3a, b). We demonstrate the effect of lymphangiogenesis through experiments on an undisclosed MFN1/2 in hLECs. MFN1/2 small interfering RNAs (siRNAs) were used to demonstrate the relationship between MFN1/2 and lymphangiogenesis in the hLECs. Effects of MFN1/2 ablation were examined in hLECs using siRNA. Each siRNA was transfected into hLECs at a final concentration of 100 nM for 2 days. Ablation of both MFN1 and MFN2 led to an approximately 93 % and 80 % reduction in MFN1 and in MFN2 protein, respectively (Fig. 3c). Next, we detected the effects of MFN1/2 ablation on tube formation. As tube formation requires cellular migration on the Matrigel, we also investigated whether attenuation of MFN1/2 affects the ability for hLECs to migrate towards HCM. As a result, tube formation reduced in the MFN1 siRNA and MFN2 siRNA group compared with control group (Fig. 3d). We have identified the critical role of MFN1/2 in a positive effect on lymphangiogenesis by HCM from the results.

### HCM promote in vivo lymphatic regeneration

**Fig. 4:**
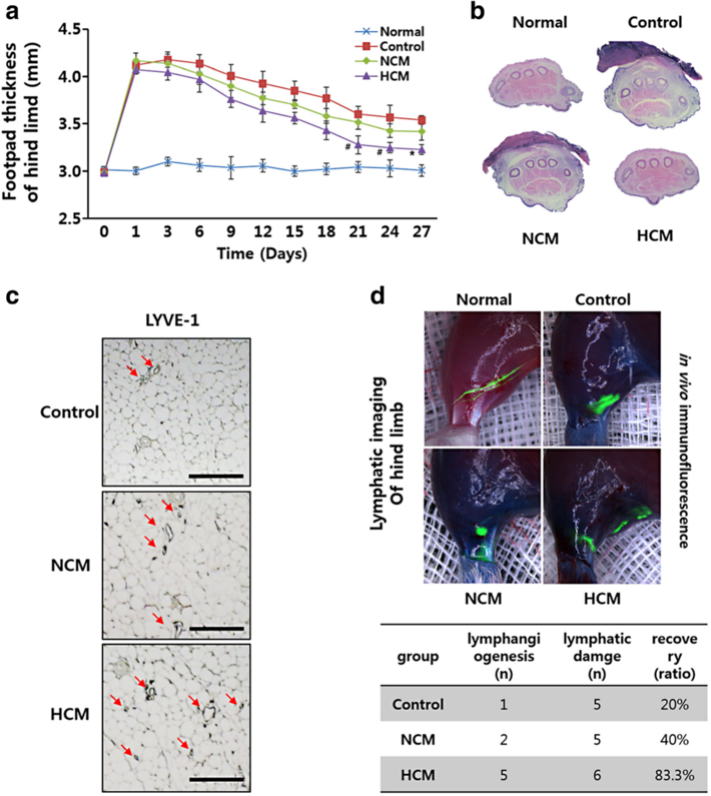
Histological analyses of lymphangiogenesis. **a** Quantitative analysis showed significantly decreased footpad thickness in each group. **b** Fibrosis of the hind limb footpad was detected with H & E stain. **c** Lymphatic endothelium marker, LYVE-1, was stained *brown* in HCM groups. Lymphatic vessels are stained *brown* (*arrows*). The scale bar represents 100 μm. **d** Fluorescence imaging of lymphatic vessels on mouse hind limbs after foot pad injection of FITC-dextran. Values are represented as the mean of three measurements with the SD indicated by error bars. *p <0.001, ^#^ *p* <0.05. *HCM* hypoxic conditioned media, *LYVE-1* lymphatic vessel endothelial hyaluronan receptor 1, *NCM* normoxic conditioned media

To confirm the ability of HCM to induce lymphangiogenesis, a mouse model was generated using electrocauterization. The maximum swelling was examined 1–3 days after electrocauterization, and the thickness of the hind limb footpad was examined every 3 days for 27 days. As shown in Fig. 4a, footpad reduction was more effective in HCM-treated groups than in NCM-treated and control groups. H & E staining showed histological edema changes, with the control group exhibiting large cytosolic damage such as fibrosis. Although footpads from the NCM-treated groups showed less cytosolic damage, HCM-treated footpads showed marked recovery compared with normal footpads (Fig. 4b). Therefore, we further investigated whether these recovery effects were due to lymphatic regeneration. Each group of footpad samples was stained with LYVE-1, a marker of hLECs, and more LYVE-1-positive staining was observed in the HCM-treated group than in the control or NCM-treated groups (Fig. 4c). To confirm the regeneration effect of HCM in hLECs in vivo, we injected FITC-dextran to visualize the lymphatic vessels. NCM also showed an increased regeneration effect on lymphatic vessels of up to twofold when compared with that of the control, whereas HCM showed a more significant lymphangiogenesis-promoting ability than the control (Fig. 4d).

## Discussion

This study demonstrated for the first time that HCM promote lymphangiogenesis by MFN1 and MFN2 regulation in hLECs. Furthermore, the study demonstrated the potential impact that lymphangiogenesis may have on damaged lymphatic vessel repair in vivo.

MSCs appear to be the most feasible, potentially safe, and effective stem cells for therapeutic lymphangiogenesis via direct differentiation toward lymphatic lineage and cytokine secretion [4, 21, 32]. Because cytokines have short-lasting efficacies, secretory modification is needed. Cytokine secretion of MSCs can be induced by hypoxia, inflammatory stimuli, and three-dimensional culture configuration. Rehman et al. [33] demonstrated that paracrine factors released from stem cells under hypoxia demonstrated anti-apoptotic or pro-angiogenic effects. Several studies have reported that HCM contain cytokines and growth factors, such as EGF, FGF-2, HGF, IGF-1, VEGF-A, and VEGF-C [34, 35]. Several reports have also stated that these cytokines could augment lymphangiogenesis in animal models of lymphedema [5–10, 36]. Our results showed higher mRNA expressions of these cytokines in hMSC under hypoxia than under normoxic conditions (Fig 2; EGF, *p* <0.001; FGF-2, *p* <0.05; HGF, *p* <0.001; IGF-1, *p* <0.001; VEGF-A, *p* <0.001; VEGF-C, *p* <0.05).

We demonstrated that HCM has the ability to enhance hLEC proliferation and migration and to increase lymphatic vessel formation. Zhan et al. also showed that culture medium from hMSCs increased lymphatic vessel formation by inducing VEGF receptor-3 (VEGFR-3)  in hLECs [37]. As shown in Fig. 2, increased mRNA expression of VEGF-A and VEGF-C by hypoxic hMSCs and HCM-treated hLECs was investigated. VEGFR-2 and VEGFR-3 are known to exist in hLECs and are activated by VEGF-A and VEGF-C, respectively [38, 39]. In addition, phosphorylation of ERK1/2 appears to be primarily caused by VEGF-A/VEGF receptor-2 upon hMSC stimulation in hLECs. Shimizu et al. [32] found that the expression of p-ERF was regulated by VEGF-C. We also showed that ERK phosphorylation and expression of LYVE-1 was increased by HCM in hLECs, which supports the increased VEGF-A expression in hLECs.

Mitochondrial function integrity is essential for organ homeostasis, particularly for rapidly dividing cells, such as hemopoietic precursor cells [40]. In this study, we ascertained the potential for HCM to promote lymphangiogenesis by regulating mitochondrial-related genes. Our results showed that mRNA expression and protein levels of MFN1 and MFN2 increased under HCM treatment in hLECs. Although the role of MFNs has been investigated in other cell types, such as endothelial cells, this is the first study, to the best of our knowledge, to investigate their roles in hLECs.

We focused on paracrine mediators of hMSCs cultured with HCM. Direct injection of HCM into a lymphedema mouse demonstrated lymphangiogenesis and edema improvement. As a precursor to future clinical trials, our study shows therapeutic potential for stimulation of lymphangiogenesis.

To use HCM, we should consider some additional details. According to a recent study [15] performed using hMSCs obtained from 20 people, there is a difference in endothelial differentiation ability of the hMSCs. Lin et al. [16] reported differences in expression of VEGF and interleukin-8 among three donors. We believe that the donor cell variation should be considered to be applicable for patient therapeutics. Furthermore, the half-life of cytokines and growth factors, which are included in the conditioned media, has been reported to be very short in vivo [41, 42]. For this reason, conditioned media must be more frequently administered. Nevertheless, HCM are a promising treatment for damaged tissue regeneration.

## Conclusions

We showed the effects of HCM from hMSCs on in vitro and in vivo lymphangiogenesis. Our results demonstrated an increase in hMSC lymphangiogenic cytokines, which can lead to proliferation, migration, and tube formations in hLECs, thereby showing hMSCs derived HCM potential therapeutic effects in the lymphedema mouse model. In addition, expression of MFN1 and MFN2 is suggested for their possible roles in lymphangiogenesis. Treatment with HCM may thus be a therapeutic strategy for lymphedema.

## Abbreviations

Ct: Threshold cycle
EBM: Endothelial basal medium
EGF: Epidermal growth factor
ERK: Extracellular signal regulated kinase
FITC: Fluorescein isothiocyanate
FGF: Fibroblast growth factor
GAPDH: Glyceraldehyde 3-phosphate dehydrogenase
HCM: Hypoxic conditioned media
H & E: Hematoxylin and eosin
HGF: Hepatocyte growth factor
hLEC: Human lymphatic endothelial cell
hMSC: Human mesenchymal stem cell
IGF: Insulin-like growth factor
LYVE-1: lymphatic vessel endothelial hyaluronan receptor 1
MFN: Mitofusin
MSC: Mesenchymal stem cell
NCM: Normoxic conditioned media
PBS: Phosphate-buffered saline
ROS: Reactive oxygen species
SD: standard deviation
siRNA: Small interfering RNA
VEGF: Vascular endothelial growth factor
VEGFR: Vascular endothelial growth factor receptor
